# Family-focused obesity prevention program implementation in urban versus rural communities: a case study

**DOI:** 10.1186/s12889-021-11967-3

**Published:** 2021-10-22

**Authors:** Colleen Flattum, Sarah Friend, Melissa Horning, Rebecca Lindberg, Jennifer Beaudette, Jayne A. Fulkerson

**Affiliations:** 1 Division of Epidemiology and Community Health, School of Public Health, University of Minnesota, 1300 S. 2nd St., Suite 300, Minneapolis, MN 55454 USA; 2 School of Nursing, University of Minnesota, Minneapolis, MN USA; 3 Minneapolis Heart Institute Foundation, Minneapolis, MN USA

**Keywords:** Children, Rural, Intervention, Obesity, Community

## Abstract

**Purpose:**

Despite public health efforts to reduce childhood obesity, there remains an unequal distribution of obesity among rural and urban children, with higher rates in rural areas. However, few studies have compared differences in program delivery. This paper aims to describe differences between an urban and rural program delivery of a family-focused, community-based intervention program to prevent and reduce obesity among children.

**Methods:**

This paper uses a case study format to provide a descriptive analysis of similar obesity prevention programs, designed by the same research team, implemented in Minnesota in different settings (i.e., an urban and rural setting) with significant community engagement in the adaptation process. The rural NU-HOME program is compared to HOME-Plus, an urban family-based obesity prevention program for school-aged children.

**Results:**

Community engagement in the adaptation process of an urban program to a rural program confirmed some anticipated program content and delivery similarities while identifying key differences that were necessary for adaptation related to engagement with the community, recruitment and data collection, and intervention delivery.

**Discussion:**

When adapting research-tested programs from urban to rural areas, it is important to identify the modifiable behavioral, social, and environmental factors associated with obesity to ensure the content of effective childhood obesity prevention programs is relevant. Customizing a program to meet the needs of the community may increase reach, engagement, and sustainability. In addition, long-term dissemination of a tailored program may significantly reduce childhood obesity in rural communities and be implemented in other rural settings nationally.

## Introduction

Children living in rural areas have about 20–25% higher odds for overweight/obesity compared to those living in urban areas of the United States [1–5]. In fact, while nationally 32% of children are overweight, the rate among rural children is 39% [6–9]. Children who are overweight are at high-risk for continued obesity and for heart disease, diabetes, cancer and osteoarthritis as adults [3, 10–12].

Because of the higher percentages of obesity in rural areas and since childhood obesity prevention programs are scarce in rural settings [12–16], more research is needed to test family-focused, obesity-prevention interventions in rural communities. When translating research tested programs from urban areas to rural areas, it is imperative to identify the specific modifiable behavioral, social, and environmental factors (e.g., geography, food availability, transportation) associated with obesity in a given community to ensure the content of effective childhood obesity prevention programs is relevant [17–21]. Furthermore, while tailored content is a well-known and essential aspect of research intervention programs [22–27], it is critical to consider other research implementation or logistical differences that may exist between urban and rural communities [16].

There tends to be a large gap between knowledge generated through research and the generalizability of that research to other community settings [23, 28–33]. Too often, researchers do not translate or disseminate their research for use in other settings where it is likely to have positive impacts. For example, translating research from urban to rural areas may be an important goal, but best practices for such translation are lacking. Therefore, the aim of this paper is to use a case study format to provide guidance and describe important program content and logistics differences (e.g., recruitment methods, data collection methods, and consideration of a “rural lifestyle” (seasonal activities)) that may require consideration when translating family-focused, childhood obesity prevention interventions developed in an urban setting to a rural setting.

## Methods

The Healthy Home Offerings via the Mealtime Environment (HOME) Plus randomized controlled trial tested the impact of a dynamic intervention program aiming to prevent childhood obesity in a metropolitan area of Minnesota in 2011–2014. The intervention program was developed to actively engage school-age children (8–12 years old) and their parents to promote regular family meals and teach cooking skills and healthful eating as outlined in Table 1. The HOME Plus study design, methods, eligibility, data collection information, and intervention description are published in detail elsewhere [34–37]. The urban HOME Plus intervention, delivered in a rural setting, produced a promising reduction in excess weight gain for those that had not started puberty [38]. Meanwhile, the Heart of New Ulm Project (HONU), a population-based demonstration project designed to reduce modifiable cardiovascular (CVD) risk factors in adults was underway in the rural community of New Ulm, MN approximately 95 miles from the Minneapolis metropolitan area [39–41]. Through university-community networking, these two groups decided to extend the adult-focused rural community programming of HONU to include child- and family-focused obesity prevention programming due to the request of the community. Thus, these two groups collaborated to write and receive funding from the National Institutes of Health to translate the urban-based HOME Plus program to a rural Minnesota community in a randomized controlled trial called the New Ulm at HOME (NU-HOME) study in 2016–2019.

**Table 1:**
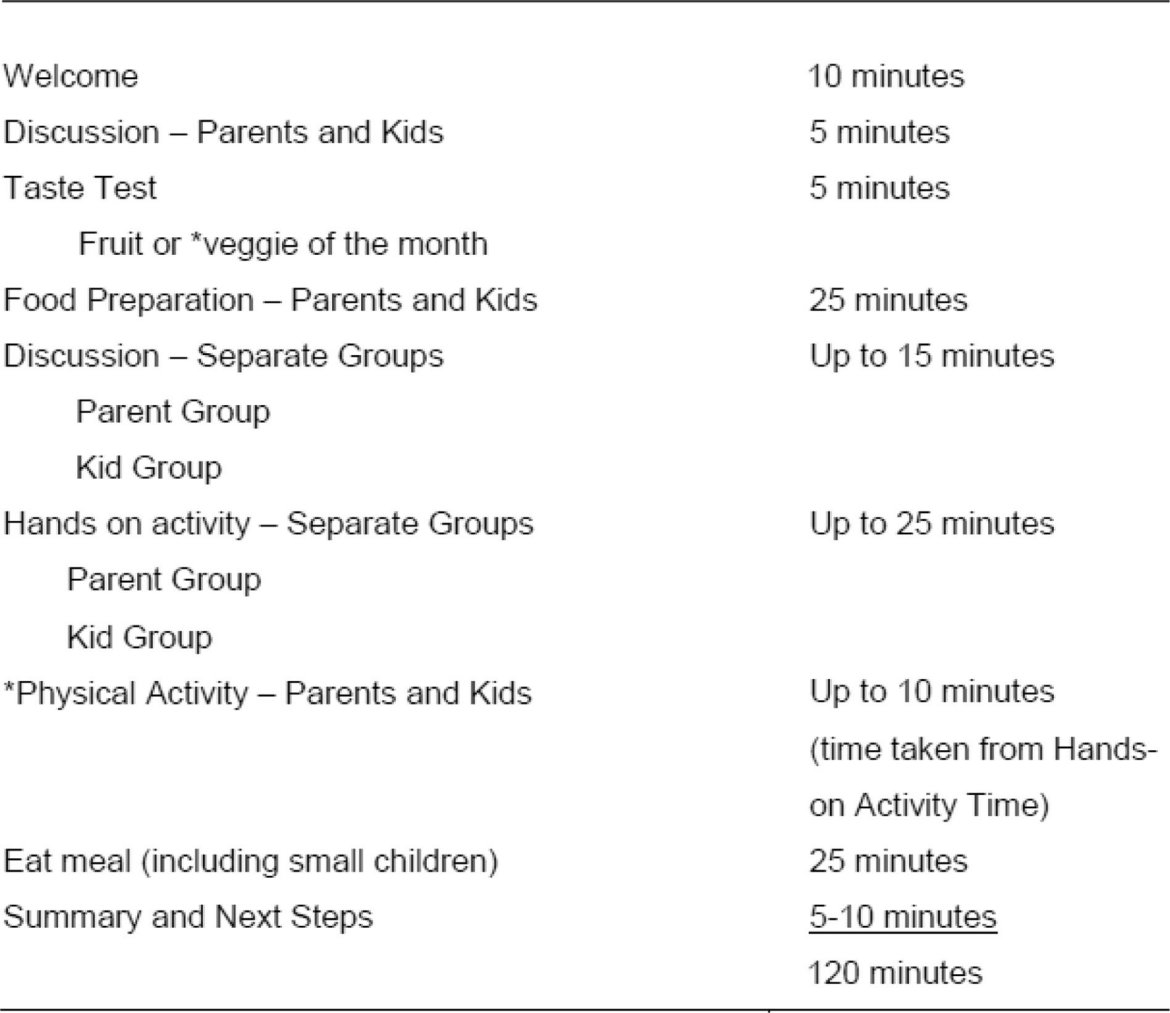
Session-At-A-Glance: Intervention Program Activities for the Urban HOME Plus and Rural NU-HOME Studies

*part of rural NU-HOME program only, physical activity and taste testing of vegetables only.

Like the urban HOME Plus study, the primary objective of the rural NU-HOME study was to prevent childhood obesity. Although considerable preliminary work with community partners was conducted for both the urban and rural settings, the collaborative effort between the HONU team and UMN researchers allowed for a deeper understanding of the unique needs of a rural environment [42]. To adapt the intervention, the NU-HOME research team worked intensely with a NU-HOME Community Action Team (i.e., staff from the local health system, schools, public health departments, and other primary stakeholders who were already engaged with community health improvements through the HONU programs). These community stakeholders were consulted about the greatest needs of the community to ensure the program would have the highest potential for impact. Community Action Team members worked to create and revise a logic model that guided program revisions. The Community Action Team met bimonthly to discuss recruitment, retention, and the intervention content and focus.

The resulting session activities followed the same format for both the urban HOME Plus and rural NU-HOME studies (Table 1). The intervention program content was also generally quite similar with the exception of physical activity, which was added for the rural NU-HOME program (Table 2). While the program content was similar in both interventions, in NU-HOME, a few modifications were made, including reduced monthly sessions from 10 to 7 (to fit within the academic school year), and intervention messages were consolidated and reinforced throughout the program (Table 2). Both programs invited all participating family members to attend the sessions, and childcare was provided for younger siblings (2–6 year olds) to remove barriers to participation. The NU-HOME study targeted a younger population of 7–10 year old children and their parents, given the importance of pubertal timing for the HOME Plus outcomes.

**Table 2:**
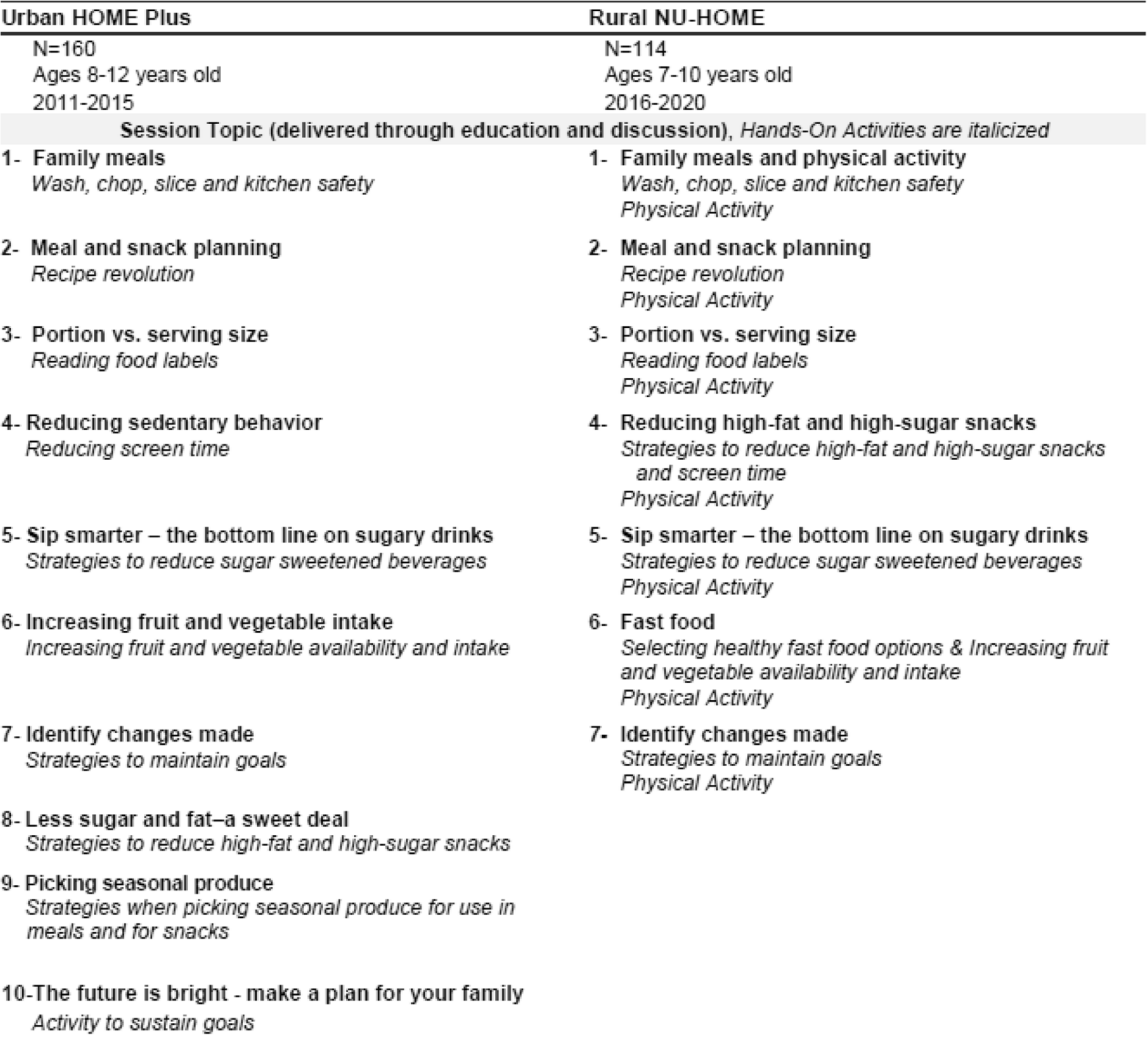
Urban HOME Plus and Rural NU-HOME Intervention Session Content

To facilitate program adaptation of an urban obesity prevention intervention to a rural community, the research team had multiple meetings with community partners to increase the likelihood of a successful trial in the rural setting. Key changes were documented in meeting minutes, observations made in the field, process data collection (e.g., number of children in childcare), and the intervention delivery guide. The following case study describes program content, logistic, and delivery adaptations that were processed with community partners and describes how the rural program differed from the urban program in the end. We review these differences deemed specific to working in a rural community below in order to support future research in rural communities to prevent childhood obesity.

## Findings

### Research adaptations for the rural NU-HOME program

#### Engagement with the community

Although the NU-HOME study research team and collaborators anticipated some changes in the delivery of an obesity prevention program in a rural setting based on HOME Plus study findings [35, 37, 38] and existing literature regarding implementing interventions in rural communities [43, 44], it was necessary to engage with the NU-HOME Community Action Team to verify or reject these anticipated changes as well as explore other modifications. Table 3 depicts logistical modifications that were discussed as we adapted the program from an urban to rural setting.

**Table 3:**
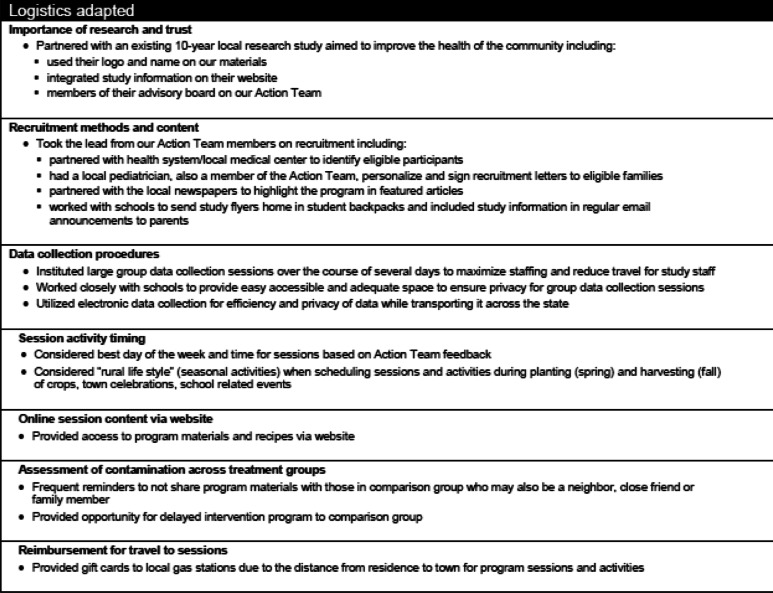
Logistical Considerations for Adapting a Family-Focused Obesity Prevention Intervention from an Urban to Rural Setting

Local support from key-stakeholders is essential for any community-based program [45–48]. In a tight-knit rural community, however, it is especially important to get buy-in and support from the community up front in order to engage and meet the needs of the community [30, 49, 50]. A large percentage of the work during the first year of the rural NU-HOME grant was focused on fostering and building relationships with key stakeholders in the rural community and getting their input on logistics and relevant intervention content [42].

The NU-HOME study research team built upon the existing HONU community partnerships. Finding and working within existing community working groups and, when necessary, establishing new working groups was instrumental in program success. Existing relationships with community partners [HONU] helped facilitate referrals to the study, aided in recruitment, and assisted with providing locations to hold data collection activities and intervention sessions.

#### Intervention content

Key session topics from the HOME Plus study intervention were presented and discussed with the Community Action Team using interactive assessments such as rating topic importance and prioritization and review and revision of a logic model. Without exception, all topics were deemed necessary for the adapted NU-HOME program. As shown in Table 2, session content for both programs was similar with a focus on family meals, the home food environment, portion sizes (of energy-dense foods) at meals and snacks, the healthfulness of family meals, self-efficacy for meal planning, cooking skill development and sedentary behaviors/screen time, all logical targets for obesity prevention. Needed changes in program content for rural NU-HOME included an added physical activity component due to reliance on automobiles to meet transportation needs, rather than biking or walking, and the evidence for multipronged interventions in obesity prevention science [51], development of a website for additional accessibility to study materials and supplemental information, and increased synergy with existing community programs and resources with the promotion of local and free events. Lessons learned from the HOME Plus study also indicated a focus on vegetables only for taste testing rather than fruit, removal of dessert (even fruit-based desserts) at sessions, targeted discussions of limited access to healthy and affordable food, and resources on food canning and storage relative to quantity and quality of available food and financial resources. Given the consistency of program content between the urban and rural programs, the focus of this manuscript is on the logistical program delivery changes that were necessary to successfully implement the trial in an effort to inform future research.

#### Recruitment and data collection

Community partnerships within the rural setting were essential to successful recruitment and data collection implementation of the NU-HOME trial. In particular, the primary health care system, schools, public health department and other stakeholders convened regularly and were highly engaged in community health improvements through HONU and we extended this engagement to the NU-HOME study activities. Common recruitment methods for family-based programs (e.g., fliers at community centers, health clinics, schools) were met with support from our community partners. This acceptance was most likely because the previous HONU program work paved the way for a greater understanding of research, expectations, etc. The rural NU-HOME study was able to build on these established and trusted relationships, which enhanced recruitment efforts. For example, direct contact between health care providers and potential study participants (through a letter and at in-person appointments) helped considerably with over half of enrolled parents reporting they heard about the study from their health care provider. The local newspapers in the two participating communities (with a large readership) ran an article about the study free of charge, and the school superintendents at several area schools gave approval to send notices home in students’ backpacks and through weekly communications with parents without extensive delays. This community buy-in, support and network allowed for an efficient, effective and timely recruitment process. In addition, the school superintendents and principals offered free use of space for data collection, intervention sessions and supply storage, which is less common in more urban programs.

Members of the New Ulm rural community were accustomed to data collection and survey completion since they had experience and familiarity with the HONU project. Yet, challenges were present in the rural community that had not occurred in our urban settings. Terms and phrases were different between the urban and rural settings that were relevant for our family meals-focused study. For example, “dinner” in an urban setting is widely accepted to mean “the evening meal.” However, in the rural setting with the NU-HOME trial, many community members referred to the midday meal as “dinner” while “supper” was the term for the evening meal. Given that the rural NU-HOME intervention focused on promoting family meals, and in particular, the family evening meal, it was critical to adopt these local terms/phrases in research surveys and intervention delivery for clarity. In addition, in the urban HOME Plus study, only one child per family was eligible for data collection participation and the average number of children per household was two, with typically only one child in the eligible age range. For the rural NU-HOME study, again only one child per family was eligible for data collection participation. However, during the feasibility assessment of potentially eligible children for the rural NU-HOME study (one per participating family), estimates of potentially eligible children from school enrollment figures were overestimated, as children in the rural communities had more siblings and many families had multiple children in the eligible age range. This resulted in two challenges: 1) the need for a protocol for selecting one child in a family when more than one was eligible, and 2) a more restricted pool of families to conduct recruitment that affected meeting recruitment goals. The latter challenge required the research team to expand to several nearby rural communities to meet recruitment goals.

#### Intervention delivery

##### Transportation

When community-based intervention programs are offered in an urban setting as in the urban HOME Plus trial, it is common to offer transportation assistance to data collection activities and intervention sessions with bus passes or by providing taxi service. However, in this (and many) rural settings, no alternate methods of transportation were available (e.g., no city buses or taxis were in service within the rural communities). Thus, the research team did not have many options for providing transportation assistance. Yet, participating families were used to these transportation norms, and once the sessions began, families connected with one another to arrange carpools, if needed. A different transportation issue was also evident when we expanded recruitment beyond New Ulm, MN to include surrounding communities. Rather than living within walking distance or relatively close to our data collection and intervention sites, many of these families had to drive up to 30 min/miles one way to attend data collection activities or intervention sessions. In order to help alleviate the burden of driving such a distance to participate in the intervention, a $10 gas gift card was given to each family for each session to help offset transportation costs.

##### Weather

The longer drives also created challenges in the Midwest winter, as traversing a few blocks or miles in poorer weather conditions in urban areas is different from traveling 30 min/miles on open road rural areas. In fact, not one urban HOME Plus study intervention session over two years was rescheduled due to weather, while the rural NU-HOME trial had four sessions canceled and rescheduled due to weather. To guide decisions on when cancellations were appropriate, we followed the lead of the local school district and canceled sessions when school activities were canceled due to weather; this protocol was easily communicated and understood by participating families. To address potential barriers around transportation and weather, we created a rural NU-HOME website with study materials and supplemental information for each session that families could access. Although there was not tremendous activity on the website, it offered an alternative method to receive materials if needed [52], [42].

##### Grocery shopping

Each NU-HOME program session included preparing a full meal for all who attended (see Table 1) including entrees and multiple side dishes, requiring a large amount of groceries for each session in both HOME Plus and NU-HOME trials. Grocery delivery is commonly available in urban settings and was used for the urban HOME Plus study. In contrast, grocery delivery was not an option for the rural NU-HOME study as was identified through community-based collaborations early in the process. Alternative arrangements were therefore made with a local grocery store that included sending the grocery list in advance and then interventionists picked up the groceries after they were assembled by store staff. In addition, not all rural communities have access to grocery stores that sell high quality ingredients such as fresh produce at reasonable prices. While the direct New Ulm community had grocery store access with high quality ingredients at reasonable prices, another neighboring community involved in the program did not have access to these resources. Therefore, groceries were often purchased in the New Ulm community for sessions delivered in a nearby community. These resources issues had direct implications for study staff workload and program logistics.

##### Family size

As mentioned previously, the rural NU-HOME study families had more children than the families in the urban HOME Plus study, which affected intervention delivery. Urban HOME Plus intervention sessions typically had 5–6 participating families, (e.g., one parent, primary child, maybe a sibling) for a total of 15–18 people; however, with larger family sizes in the rural NU-HOME study, sessions frequently had up to 24 attendees per session. As a result, the kitchen space in the rural community needed to be larger, the interventionists need to adapt to engaging more people at one time, and childcare was more heavily utilized at each of the sessions for siblings aged 2–6 years old when compared to the urban setting.

##### Local events and customs

Compared to an urban setting, in the rural setting of the NU-HOME trial, community events often drew a larger proportion of community members, including whole families. For example, school plays, football games, town celebrations (e.g., Octoberfest) and even funerals greatly influenced attendance at NU-HOME intervention sessions, as most of the participants attended the same schools and the communities were very connected. In addition, farming schedules, hunting season, teacher conferences, and religious events were a priority for many families, and it was necessary for the research team to carefully avoid these dates when scheduling study related activities. These events were not as logistically challenging when scheduling the HOME Plus sessions in the urban community.

##### Treatment group contamination

In the rural NU-HOME study, the community was smaller than the urban community setting of the HOME Plus study. As such, the smaller community often meant that participants were neighbors, coworkers, close friends or relatives, and therefore, there was an increased risk for contamination (e.g., intervention participants sharing session information with control participants) which could lead to unintended behavior change among control group members. Therefore, intervention staff discussed with families in the intervention group the importance of the randomization process and reiterated the importance at each session not to share information with those outside of the intervention group during the trial.

##### Delayed intervention programming

As part of the urban HOME Plus study design, participating families randomized to the control group received a monthly newsletter. In early discussions with members of the rural community, there was less acceptance among stakeholders of randomization to a “newsletter” control group. Community stakeholders believed all families would be interested in participating in the rural NU-HOME hands-on family cooking and physical activity program. Therefore, the study design was adapted in the funded grant proposal to include a “waitlist control” (e.g., the comparison group would participate in intervention sessions upon completion of the study). The delayed intervention required significantly more funding, staffing, and organization than delivering newsletters, but it was deemed essential for study success.

##### Recipe selection and adjustments to reduce costs

In both the urban and rural settings for the HOME Plus and NU-HOME studies, respectively, efforts were made to be mindful of ingredient availability and cost when selecting recipes to be promoted in the intervention program. Thus, session recipes included as many seasonal fruits and vegetables as possible and did not include items with limited availability. These considerations allowed families to purchase recipe ingredients at the local farmer’s market(s) or use produce from community or personal gardens.

Differences in cost between urban and rural settings were also seen for mileage and fuel cost for study staff. While some study staff already lived and worked in the rural communities where the NU-HOME study took place, some staff needed to drive from urban areas to deliver the intervention and conduct data collection. These were additional study expenses. Considerable travel costs need to be considered when conducting an intervention in a rural setting if study staff need to travel further distances to conduct study business (i.e., they do not live in or nearby the community given the location of the home institution/university).

## Discussion

Review of recent literature reveals that rural children are at greater risk for obesity than their urban peers [6, 15] and previous studies involving rural families have been limited in scope and not specifically designed for the needs, interests, benefits and concerns of rural families [14, 17, 18, 53]. Rural families face a number of unique challenges in an attempt to maintain behaviors to promote health and prevent obesity [54]. Such potential challenges include accessibility (e.g., lower availability of fresh fruits, vegetables and other recipe ingredients; travel distances to major grocery stores, exercise facilities, hospitals and clinics, schools and even neighbors are often great distances from their homes), lower incomes as many rural families have limited or seasonal earnings, and limited access to high-speed communications [55]. Therefore, to be an effective obesity prevention program in rural communities, intervention programs must be developed with these potential challenges in mind and must be tailored to the local context and setting. It is important to note that much of the intervention content did not change when adapting our childhood obesity prevention program from an urban setting to a rural setting (likely because the drivers of obesity are similar across settings); however, there were many important logistical changes needed in program delivery for the rural setting.

Effective obesity prevention programs for rural families demand unique recruitment and program delivery methods. First, collaboration with key-stakeholders is necessary in order to engage and meet the needs of the community. Stakeholders are particularly well suited to provide insight since they live in the community and are intimately familiar with their residents, including their needs, interests and the concerns as those noted above. Second, the communities participating in the rural NU-HOME study had experience with successful recruitment and data collection implementation due to the previous groundwork of the HONU program that paved the way for research in this community. Third, the community-based NU-HOME program targeted changes in the places where families live, learn, work, and play and provided multiple levels of influence including individual behaviors, family settings, and community institutions.

Given the research considerations related to NU-HOME program delivery within a rural setting, guided by the HONU project and the urban HOME Plus study, we delivered a unique family-focused intervention designed specifically for rural families. By developing our NU-HOME program, our hope is that this intervention has the potential to be applied and broadly implemented throughout rural communities to increase knowledge and build skills that could lead to behavior change and eventually health equity for rural youth. The rural NU-HOME program also showcases important differences in delivering a family-focused, community-based obesity prevention program from an urban to rural setting.

While this case study has several strengths in how it showcases important differences between translating an urban program to a rural community, a limitation of this work is that an implementation science framework was not applied and could have provided excellent structure for program adaptations. Unfortunately, the timing of NU-HOME study funding and adaptation of the intervention program from the urban HOME Plus study was occurring right before the implementation science field was just coming into its prime. Thus, although our adaptation work was based on existing high-quality translation methodologies, we recommend future intervention adaptations use an implementation science framework for guidance [33, 56–58]. We believe our case study approach informs the field moving forward by providing a base of information for program adaptations for families living in rural communities given the dearth of literature in this area to date.

Public health agencies may find that by forming community joint ventures with schools, healthcare groups, local private sector businesses, and the community, they can focus their mission and create opportunities for healthy living and reduce obesity in rural communities. No single intervention alone will resolve the problem of rural community obesity. However, when a multi-phase approach of activities and programs are offered together, they can encourage and develop lifestyle modifications that encourage healthy behaviors and reduce rural obesity. The collaboration of our research team with existing community organizations that had a similar health promotion mindset, goals, and infrastructure development provides an excellent model for future interventions in rural communities.

This paper highlights specific differences in program delivery between urban and rural community engagement, recruitment and data collection, and intervention programming. This information is particularly useful for others in the early stages of introducing obesity prevention interventions and is broadly informative for programs and funders working in rural areas or those who wish to translate programs developed in urban areas to rural areas.

## Data Availability

The data analyzed during the current study is available from the corresponding author on reasonable request.
